# Stria terminalis, amygdala, and temporoparietal junction networks facilitate efficient emotion processing under expectations

**DOI:** 10.1002/hbm.24779

**Published:** 2019-08-28

**Authors:** Ilvana Dzafic, Lena Oestreich, Andrew K. Martin, Bryan Mowry, Hana Burianová

**Affiliations:** ^1^ Queensland Brain Institute University of Queensland Brisbane Australia; ^2^ Centre for Advanced Imaging University of Queensland Brisbane Australia; ^3^ Australian Research Council Centre of Excellence for Integrative Brain Function Australia; ^4^ University of Queensland Centre for Clinical Research Brisbane Australia; ^5^ Department of Psychology Durham University Durham UK; ^6^ Queensland Centre for Mental Health Research Brisbane Australia; ^7^ Department of Psychology Swansea University Swansea United Kingdom

**Keywords:** amygdala, emotion perception, prior expectations, stria terminalis, temporoparietal junction

## Abstract

Rapid emotion processing is an ecologically essential ability for survival in social environments in which threatening or advantageous encounters dynamically and rapidly occur. Efficient emotion recognition is subserved by different processes, depending on one's expectations; however, the underlying functional and structural circuitry is still poorly understood. In this study, we delineate brain networks that subserve fast recognition of emotion in situations either congruent or incongruent with prior expectations. For this purpose, we used multimodal neuroimaging and investigated performance on a dynamic emotion perception task. We show that the extended amygdala structural and functional networks relate to speed of emotion processing under threatening conditions. Specifically, increased microstructure of the right stria terminalis, an amygdala white‐matter pathway, was related to faster detection of emotion during actual presentation of anger or after cueing anger. Moreover, functional connectivity of right amygdala with limbic regions was related to faster detection of anger *congruent with cue*, suggesting selective attention to threat. On the contrary, we found that faster detection of anger *incongruent with cue* engaged the ventral attention “reorienting” network. Faster detection of happiness, in either expectancy context, engaged a widespread frontotemporal‐subcortical functional network. These findings shed light on the functional and structural circuitries that facilitate speed of emotion recognition and, for the first time, elucidate a role for the stria terminalis in human emotion processing.

## 1 INTRODUCTION

In a dynamic social environment, the ability to quickly recognise a potential friend or threatening foe is essential for survival. In social situations in which, for example, threat is expected, allocation of attention toward aggressive cues to enable a faster response is paramount (Adolphs, 2013; Barbalat, Rouault, Bazargani, Shergill, & Blakemore, 2012). Conversely, in seemingly innocuous social situations, threat may appear unexpectedly, requiring a rapid shift of attention toward aggressive cues (Ohman, Lundqvist, & Esteves, 2001; Schmidt‐Daffy, 2011). Identification of the neural circuitry, facilitating rapid recognition of realistic, dynamic representations of emotion in the healthy brain, is essential for our understanding of aberrant neural connectivity in pathologies characterised by impaired emotion processing, such as schizophrenia, major depression, or anxiety (Barkl, Lah, Harris, & Williams, 2014; Chan, Li, Cheung, & Gong, 2010; Demenescu, Kortekaas, den Boer, & Aleman, 2010). Therefore, in the current study, we investigated the neural networks underlying the fast recognition of happy and angry emotional expressions, in conditions where these emotions were either congruent or incongruent with prior expectations (induced by an explicit cue). For this purpose, we examined the functional networks that subserve rapid response to emotions presented in a dynamic, audio‐visual environment. Critically, for the first time, we related these functional networks to structural networks previously linked to emotion processing, but with an unknown relationship to *emotion processing speed*.

Previous studies exploring rapid emotion processing (particularly threat) in humans and animals have provided converging evidence that implicate an amygdala network as core circuitry (Anderson, Christoff, Panitz, De Rosa, & Gabrieli, 2003; Gross & Canteras, 2012; Hooker, Germine, Knight, & D'Esposito, 2006; LeDoux, 1998; Marstaller, Burianová, & Reutens, 2016b; Reinders et al., 2006). In humans, the amygdala is found to enable rapid processing by means of selectively directing attention to relevant and salient, coarse cues (Dolan & Vuilleumier, 2003; Garvert, Friston, Dolan, & Garrido, 2014; Mendez‐Bertolo et al., 2016; Pessoa & Adolphs, 2010; Vuilleumier, Armony, Driver, & Dolan, 2003). There is evidence that the amygdala may play a central role in processing unconscious stimuli, via a subcortical route, which has been dubbed the neural “alarm” system for rapid alerting to threat (Liddell et al., 2005). However, there is debate whether this entails specifically processing threatening stimuli unconsciously, or *any* relevant stimulus (Pessoa & Adolphs, 2010; Tamietto & de Gelder, 2010; Williams, Morris, McGlone, Abbott, & Mattingley, 2004). The extended amygdala, including the bed nucleus of the stria terminalis (BNST), has been consistently found to have a role in directing attention to threatening cues (Herrmann et al., 2016; Somerville, Whalen, & Kelley, 2010; Walker, Toufexis, & Davis, 2003) and involvement in emotional face perception (Sladky et al., 2017). The stria terminalis, a white matter tract, which connects the amygdala, hippocampus, BNST, caudate, and thalamus, and which branches out to multiple septal and hypothalamic nuclei, has recently been traced in humans (Rafal et al., 2015), but its role in human emotion processing remains unknown. In the current study, our aim was to provide the first evidence for the role of the stria terminalis in human social behaviour.

Another tract, which connects the amygdala to visual regions, is the inferior longitudinal fasciculus (ILF). In a recent study, Marstaller et al. (2016b) demonstrated that increased microstructure (i.e., fractional anisotropy; FA) in the ILF is associated with faster detection of anger and fear. Despite these findings, the emotion stimuli consisted of only threat‐related expressions (i.e., positive emotions, such as happiness, were not explored), and the stimuli were presented in the form of static images of faces. The use of static stimuli in experimental paradigms fails to uncover processes involved in fast detection of realistic, dynamic emotions, and limits our understanding of the neural circuitries that underlie these processes. Crucially, compared to static expressions, dynamic emotion has been shown to rely on *predictive processes* to a greater extent, due to the uncertainty in the constantly moving and changing features (Kaufman & Johnston, 2014; Palumbo & Jellema, 2013). Prior expectations direct attention to features that are aligned with predictions (Friston, Kilner, & Harrison, 2006) and are a vital process in naturalistic emotion perception.

The functional and structural networks, associated with *expectancy‐driven* rapid detection of positive and negative emotions, remain unknown. Previous research has provided evidence for differential regional activation in the right amygdala (rAMY) and parietal cortex—in particular right temporoparietal junction (rTPJ)—for emotions congruent or incongruent with prior expectations, respectively (Bermpohl et al., 2006; Browning & Harmer, 2012; Dzafic, Martin, Hocking, Mowry, & Burianova, 2016; Ueda et al., 2003). However, how these regional differences relate to network level differences, for both functional and structural circuitry, has not been investigated. In regards to incongruency with expectations, seminal work by Corbetta, Patel, and Shulman (2008) established that “reorienting” to unexpected, but relevant stimuli (e.g., threatening human faces) involves a right ventral frontoparietal network, labelled the ventral attention network (Corbetta & Shulman, 2002). Although the ventral attention network has consistently been linked to the rapid processing of unexpected stimuli, the role of the underlying structural connections that enable this efficiency in processing have not yet been established. Regions comprising the ventral attention network are interconnected by the inferior fronto‐occipital fasciculus (IFOF) (Hattori et al., 2017), which runs within the frontal lobe, insula, and temporal stem, and connects the frontal operculum with the occipital, parietal, and temporobasal cortex (Catani & De Schotten, 2008; Chechlacz, Gillebert, Vangkilde, Petersen, & Humphreys, 2015). Damage to the right IFOF has been shown to impair emotion recognition (Philippi, Mehta, Grabowski, Adolphs, & Rudrauf, 2009), and greater FA in the IFOF has been related to better emotion discrimination (Unger, Alm, Collins, O'Leary, & Olson, 2016). Also, the speed of visual information processing has been related to hemispheric lateralization of the IFOF (Chechlacz et al., 2015). In the current study, we investigated if the IFOF has a role in the speed of processing emotions that are incongruent with cues.

The objective of the current study was to delineate the structural and functional networks underlying fast response to naturalistic emotions, in conditions congruent or incongruent with prior expectations. For this purpose, we used a previously validated Dynamic Emotion Perception (DEP) task, in which instruction cues and frequency of display were used to elicit prior expectations during viewing of angry and happy audio‐visual videos (Dzafic et al., 2016). Based on the results of previous studies, we predicted that faster recognition of anger *congruent* with cue would involve the recruitment of rAMY functional network and greater FA in the right stria terminalis and ILF‐amygdala structural networks. Conversely, we expected that faster recognition of anger *incongruent* with cue would involve the recruitment of rTPJ [a core region of the ventral attention network (Corbetta et al., 2008)] functional network and greater FA in the right IFOF‐TPJ structural network.

## 2 METHODS

### 2.1 Participants

Forty‐six healthy, right‐handed males [mean (M) age = 39.83, standard deviation (SD) = 12.04] were recruited through on‐line advertising to staff and students across the University of Queensland. A telephone interview was conducted before recruitment to ensure that participants had normal or corrected‐to‐normal vision, were not taking medication, and had no history of neurological disorders, or metal implants in their body. The cohort scored within the normal healthy range on IQ, estimated using 2 subsets (vocabulary and matrix reasoning) of the Wechsler abbreviated scale of intelligence (WASI; Wechsler, 1999; *M* = 111.65; SD = 13.48; range = 80–140).

Participants were provided with an information sheet that included a full description of the study and fMRI procedure. After reading and understanding the document, written informed consent was obtained from each participant. This research was approved by the Medical Research Ethics Committee of the University of Queensland. Participants received $30 as reimbursement.

### 2.2 Paradigm: dynamic emotion perception task

#### 2.2.1 Materials

Participants completed the DEP task (Dzafic et al., 2016) during fMRI. The DEP task involved viewing audio‐visual video clips of an actor expressing emotions congruent or incongruent with prior expectations. Prior expectations were induced by displaying an emotional instruction cue before the video clips (Barbalat, Bazargani, & Blakemore, 2013) and by having a greater number of videos congruent with the emotion in the instruction cue (Chambon et al., 2011).

**Figure 1:**
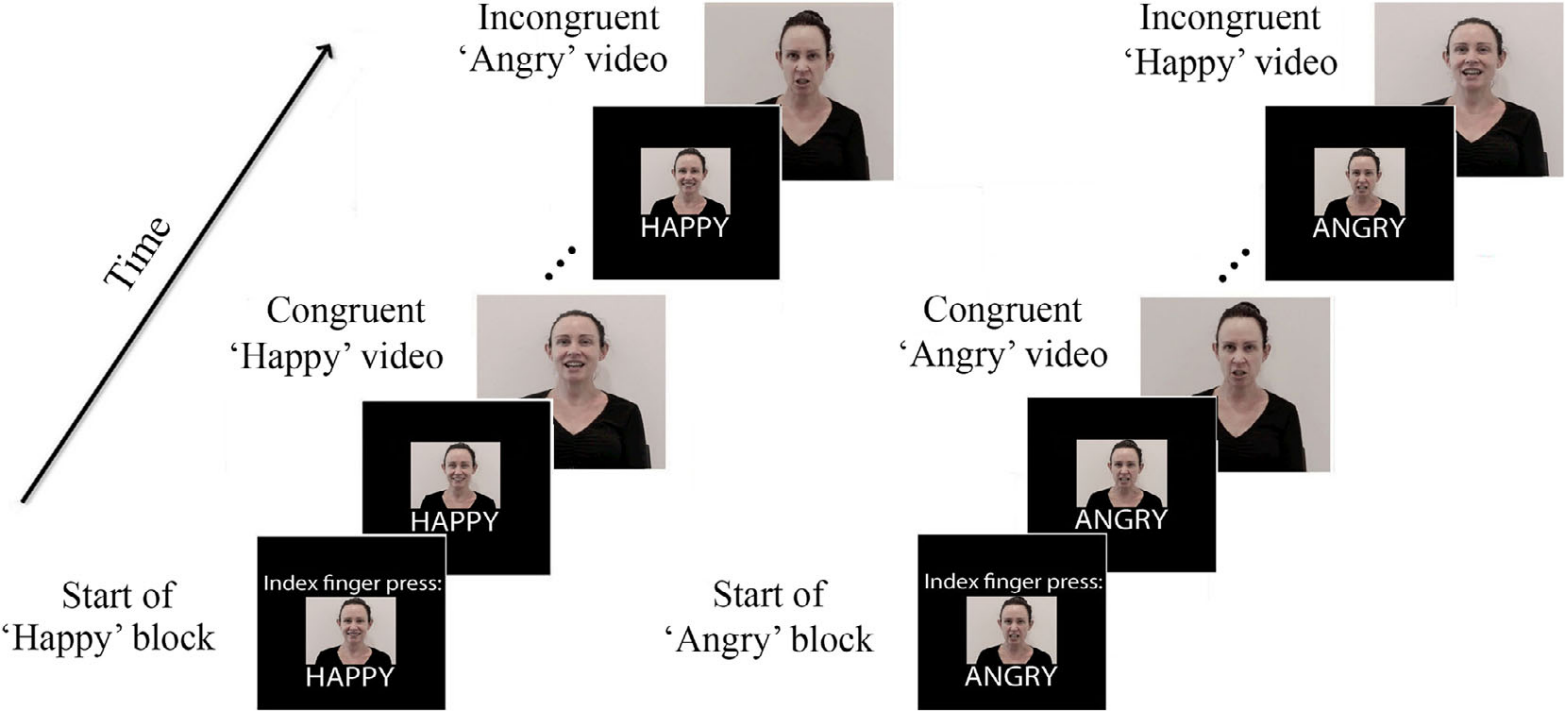
A graphical depiction of *congruent* and *incongruent* angry (threat) and happy trials within the dynamic emotion perception task. In an angry block, participants were asked to press a button with their index finger for angry videos (*angry congruent* trial) and press a button with their middle finger for happy videos (*happy incongruent *trial). However, in a happy block, participants were asked to press a button with their index finger for happy videos (*happy congruent* trial) and their middle finger for angry videos (*angry incongruent* trial)

Experimental stimuli included emotion videos, instruction cues, and emotion cues, as described below. For an in‐depth description of the development, editing, and piloting of these stimuli, please see Dzafic et al. (2016). (1) Emotion videos: there were 48 emotional videos in total, 16 for each emotion condition: angry, happy, and neutral. The videos presented a female actor speaking 16 different sentences, which were emotionally ambiguous (i.e., the semantic content made sense for multiple emotions). By keeping the content constant across the three emotions, we controlled for linguistic confounds. (2) Instruction cue: the instruction cue contained a still picture of the actor expressing an emotion (either angry, happy, or neutral), with the expressed emotion written in white text underneath the picture. The picture and the writing were presented in the centre of the cue and overlaid on a black background. Above the picture, the instruction cue contained white text, instructing the participant to make an “index finger press” for the emotion in the picture (see Figure 1). (3) Emotion cues: the emotion cues were identical to the instruction cues, except that they did *not* contain the text above the picture to make an “index finger press.”

#### 2.2.2 Design

The experimental procedure consisted of three runs of the DEP task and nine experimental conditions: three cues (happy, angry, and neutral) × three emotional videos (happy, angry and neutral). When the emotion in the cues and video matched, this was a “congruent” condition, whereas when the emotion in the cues and video did not match, this was an “incongruent” condition. Please note that the task included angry, neutral, and happy video clips. However, for the purposes of this study and research question, we conducted analyses only on the angry and happy video clips, as the objective of the study was to investigate emotion perception in different expectancy contexts. The different expectancy conditions were: angry congruent (angry video preceded by angry cues; AC), angry incongruent (angry video preceded by happy cues; AI), happy congruent (happy video preceded by happy cues; HC), and happy incongruent (happy video preceded by angry cues; HI) (see Figure 1).

Within each imaging run, there were nine experimental blocks. Each experimental block began with an instruction cue (3 s), followed by six or nine trials, consisting of an emotion cue presented for 1 s, followed by an inter‐stimulus interval (ISI; a black screen presented for a mean duration of 1 s), which was followed by an emotion video presented for 3 s. The ISI was jittered within a block, with a uniform distribution between 500 ms and 1,500 ms, of either 6 × 200 ms intervals (during blocks of six videos) or 9 × 125 ms intervals (during blocks of nine videos). The reason the blocks were of different lengths was to reduce the predictability of how many trials each block contained, and thus the ability of participants to predict the number of incongruent or congruent videos. Alternating the number of videos was also done to eliminate repetitiveness, as this may result in fluctuations of attention.

The experiment was a mixed design, meaning that different emotion videos were presented in an event‐related fashion within the blocks of a specific emotion cue. In other words, within each block, the cue always carried the same emotion (e.g., angry), but the videos within that block would alternate the emotion (e.g., four angry videos, one happy video, and one neutral video). The video clips within a block were randomised using Microsoft Excel, so that the appearance of congruent or incongruent videos could not be predicted. The emotion blocks were counterbalanced between runs, as were the runs between participants, using the Balanced Latin Squares method.

### 2.3 Experimental procedure

The participants were asked to respond to the video clips by indicating if the emotion presented in the instruction cue matched the emotion expressed in the video. Specifically, participants were told to press the button with their index finger when the video clip was congruent with the instruction cue emotion or press with their middle finger when it was not. All responses occurred within 3 s during the video clips. Accuracy and reaction times (RTs) were recorded for each trial.

Before the fMRI experiment, participants were trained with a practice task outside the MRI scanner. Both the practice and fMRI tasks were presented using E‐Prime 2.0 software [https://www.pstnet.com/eprime.cfm, 2013; Schneider et al., (2012)] on a Windows computer screen. The practice task consisted of nine blocks, and feedback was given if the correct/incorrect button was pressed. The goal was to ensure that participants understood the aim of the task, and that the finger response became familiar outside of the scanner. During the fMRI experiment, the DEP task was seen by participants through a tilted mirror attached to the head coil. Responses were recorded using a custom‐built MRI‐compatible response box. Participants were instructed to respond as quickly and as accurately as possible. No feedback was provided during the actual experiment.

After the fMRI experiment, participants completed the WASI (Wechsler, 1999) in a testing room outside the MRI scanner. The study was conducted at the Centre for Advanced Imaging, University of Queensland.

### 2.4 MRI acquisition and preprocessing

Structural and functional MRI images were acquired by a 3T Siemens Magnetom TrioTim system using a 12‐channel head coil. The scans collected for each subject, in a session were as follows: localizer, T1‐weighted anatomical image MP2RAGE sequence (repetition time (TR): 1900 ms, echo time (TE): 2.32 ms, resolution: 1 mm^3^, FoV: 230 mm, 196 slices), T2* weighted echo‐planar sequence (TR: 3000 ms, TE: 30 ms, resolution: 2.5 mm^3^, FoV: 192 mm, 46 slices), diffusion‐weighted imaging [high‐angular‐resolution diffusion‐weighted imaging (HARDI) acquisition protocol; TR: 8400 ms, TE: 100 ms, resolution: 2 mm^3^ isotropic, slices: 60, FoV: 300 mm, *b*‐value: 2000 s/mm^2^, 64 directions], and resting‐state (TR: 3000 ms, TE: 30 ms, resolution: 2.5 mm^3^, FoV: 192 mm, 46 slices). The total scanning time per session was 45 min.

#### 2.4.1 Functional magnetic resonance images

Standard preprocessing of the images was carried out using Statistical Parametric Mapping (SPM8) (http://www.fil.ion.ucl.ac.uk/spm/software/spm8). The preprocessing steps were as follows: slice timing on the functional images to correct for differences in slice acquisition times within each volume, using the middle slice as reference; realignment (estimate and reslice) on the functional images, to correct for inter‐scan movement within each run (no participant was excluded for excessive movement (defined as >3 mm translation or >2° rotation); co‐registration of the functional and structural images; segmentation of the structural image, with heavy regularisation (0.1) recommended for MP2RAGE sequence; normalisation of the resliced images into a standardised, stereotaxic space (according to the Montreal Neurological Institute template); and smoothing of normalised images with 6 mm full‐width‐at‐half‐maximum isotropic Gaussian kernel.

#### 2.4.2 Diffusion‐weighted images

The preprocessing was conducted using tools implemented in MRtrix3 (Tournier, Calamante, & Connelly, 2012). Diffusion‐weighted images were corrected for head movements (Smith et al., 2004) and eddy current distortions (Andersson & Sotiropoulos, 2016), and inhomogeneities were removed (Zhang, Brady, & Smith, 2001). The response functions were estimated using the single‐fibre tournier algorithm and used to calculate the fibre orientation distributions using constrained spherical deconvolution (Tournier, Calamante, & Connelly, 2013). A deterministic tractography algorithm based on spherical deconvolution with a step size of 0.2 mm, maximum path length of 200 mm, and minimum path length of 10 mm was used to generate streamlines of the right stria terminalis, ILF, and IFOF. FA was extracted from the maps of the individual white matter pathways, which was defined by the fraction of tracks to traverse each voxel.

#### 2.4.3 Tractography

The regions of interest (ROIs) for the tractography were defined using an online FSL Harvard‐Oxford atlas. The stria terminalis was reconstructed by placing a seeding ROI in the right amygdala—the seeding ROI was dilated two times. Inclusion ROIs were also created for the right hypothalamus and thalamus/caudate, and dilated two times. An exclusion ROI was created inferior to the right caudate head to prevent the formation of the amygdafugal tract. These ROIs were then warped to individual space from MNI space and manually shaped to the individual person's anatomy with guidance from the FSL Harvard‐Oxford atlas. Tracts for the stria terminalis were indeterminable for eight participants. The IFOF was reconstructed by placing a seeding ROI in the coronal slice, anterior to the genu of the corpus callosum, delineating the majority of the cerebral hemisphere. An inclusion ROI was drawn in the superior and middle temporal gyri, and a third ROI in the occipital lobe, posterior to the splenium of the corpus callosum, on a coronal slice. For the reconstruction of the ILF, a seeding ROI was placed in the occipital lobe, posterior to the splenium of the corpus callosum, and covering the majority of the cerebral hemisphere. A second ROI was drawn in the anterior temporal lobe. For the IFOF and ILF, exclusion ROIs were drawn in the midline sagittal plane, to exclude interhemispheric projections. Further exclusion ROIs were drawn to exclude outlier tracts not consistent with the known anatomy of the pathways of interest. Examples of the stria terminalis, ILF, and IFOF tracts and ROIs used for the tractography are displayed in the Supplementary Materials (see Figures S1–S3).

## 3 DATA ANALYSIS

We used a multivariate, partial least squares (PLS) approach for the functional/structural connectivity analysis and relating behavioural performance to the delineated networks. PLS examines the relation between activity in a selected brain region and activity in the rest of the brain across task conditions, as well as the relation of these functional networks to structural networks, and behavioural performance in each experimental condition. The PLS technique allows identification of distributed patterns of neural activity, rather than the independent activity of a single brain region; thus, this analysis is optimally suited to investigating emotion perception, which engages a widespread and interactive brain network (Arsalidou, Morris, & Taylor, 2011; Vuilleumier & Pourtois, 2007).

Based on the findings of regional activations underlying congruence or incongruence with cue (Barbalat et al., 2013; Dzafic et al., 2016), we conducted an analysis in which two regions were selected: the right amygdala [18‐8‐18] and the right temporoparietal junction [60−50 34]. In our study, the selection of the regions was both data‐driven (please see Dzafic et al., 2016) and literature‐driven (Bishop, 2008; Vuilleumier & Pourtois, 2007; Marstaller et al., 2016b; Corbetta et al., 2008; Decety & Lamm, 2007; Doty, Japee, Ingvar, & Ungerleider, 2014). We conducted two separate analyses to identify the correlation between activity in the (1) rAMY with activity in the rest of the brain, right stria terminalis FA values, right ILF FA values, and behaviour; and (2) rTPJ with activity in the rest of the brain, right IFOF FA values, and behaviour. Our behavioural measure in each analysis was accurate RTs from each participant in the congruent/incongruent, happy/angry conditions. Three participants were removed from the final analyses due to being outliers (*z*‐score > 3).

The procedure for the functional connectivity analysis involved extracting the blood‐oxygen‐level dependent (BOLD) values from the two regions, from the onset of each angry/happy video, across 6 time points (TRs), as this time period captures the hemodynamic response function. The averaged activity for each region, as well as the mean FA values and mean RTs (the functional, structural, and behavioural variables), were then correlated with activity in all other brain regions, across all participants, and within each experimental condition, to form a covariance matrix. Next, the covariance matrix is decomposed with singular value decomposition (SVD), resulting in a set of orthogonal variables (latent variables; LVs). Each LV consists of three components: singular values (significance for a given LV), voxel saliences (spatiotemporal activity for a given LV), and task saliences (degree to which each condition is related to the brain‐variable correlations within the given LV). Finally, the significance for each LV was determined by conducting 500 permutations (McIntosh, Bookstein, Haxby, & Grady, 1996). Corrections for multiple comparisons are not necessary in PLS, as the voxel saliences are calculated in a single mathematical step on the whole brain. For each LV, “brain scores” are computed for each participant, which indicate the degree to which each participant shows the pattern of brain activity identified, across conditions. We calculated the correlation between the brain scores from each significant LV and the rAMY/rTPJ BOLD values to assess the relation between the whole‐brain pattern and activity in the two regions. The robustness of the voxel saliences was assessed with bootstrap estimation of the standard errors (SE), with 100 iterations. Bootstrap ratios are obtained by dividing each voxel's mean salience by its bootstrapped SE. Peak voxels with a bootstrap ratio > 3.0 and cluster size of 100 or more voxels were considered, as this approximates *p* < 0.001. The results display the Pearson product–moment correlation coefficient between whole‐brain scores and other variables (e.g., the RTs, FA values, and rAMY/rTPJ percent signal change) for each condition. These correlations reflect the three‐way relation between behaviour, structure, and function.

## 4 RESULTS

### 4.1 Structure–function–behaviour relation among right amygdala, stria terminalis, and ILF

The analysis with right amygdala identified two statistically significant LVs (*p* < 0.01), accounting for 20.53% and 12.97% of covariance in the data.

#### 4.1.1 LV1: right amygdala network, ILF, and faster response to happy emotions

**Figure 2:**
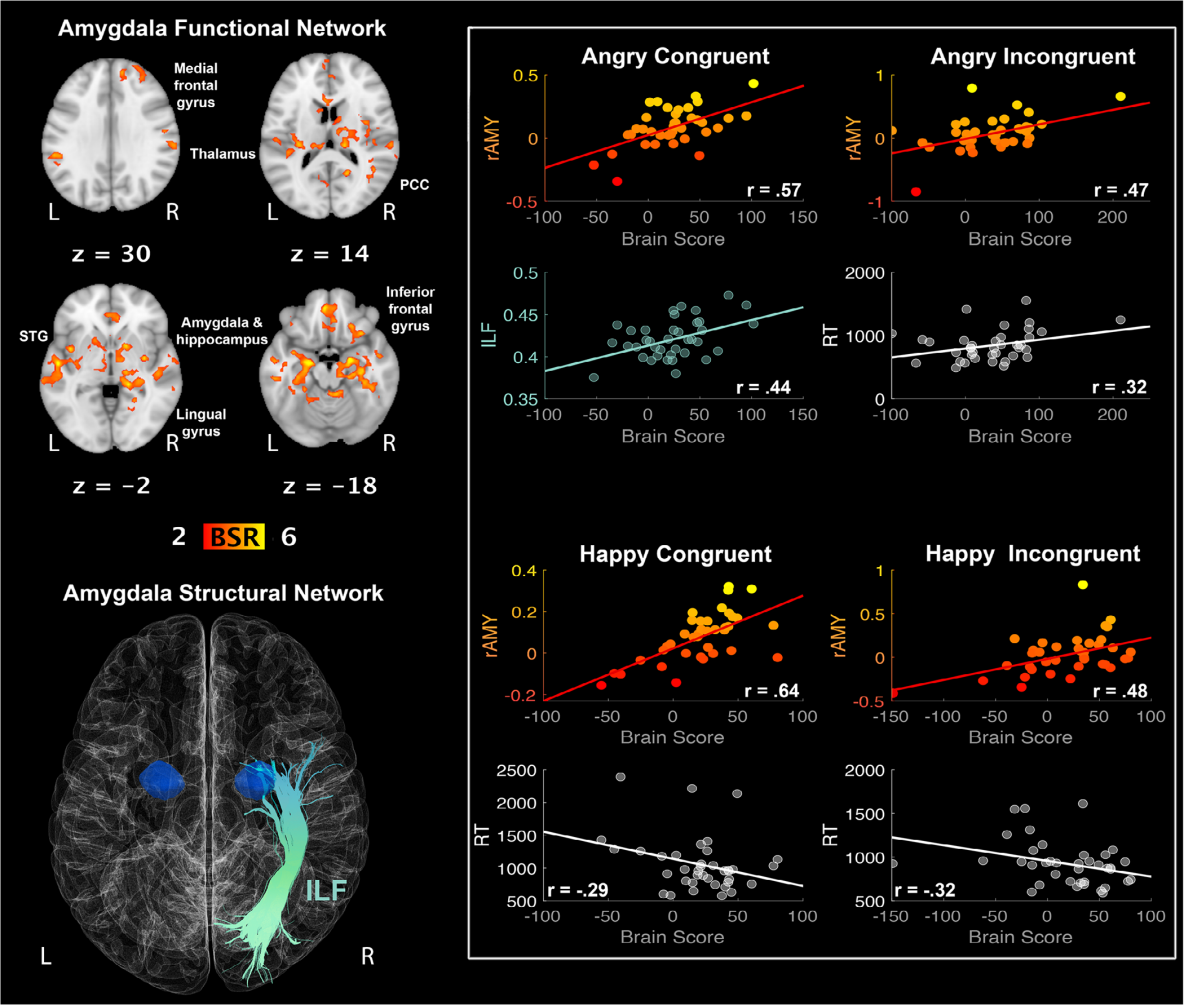
LV1: functional connections with right amygdala (rAMY) and structural/behaviour PLS results. (Top left panel) A pattern of correlated whole‐brain activity, (bottom left panel) structural network: right inferior longitudinal fasciculus (ILF). (Top right panel) During angry conditions, correlations between activity in rAMY seed and brain scores representing network activity displayed in the top left panel, with positive correlation for ILF during congruent conditions, and positive correlation for reaction times during incongruent conditions. (Bottom right panel) During happy conditions, correlations between activity in rAMY seed and brain scores representing network activity displayed in the top left panel, with negative correlation for reaction times during congruent and incongruent conditions. rAMY seed activity is displayed in percent signal change and reaction times are displayed in milliseconds. BSR threshold is set at 2 (*p* < 0.05), for visualisation purposes; however, reported whole‐brain activity is set at BSR 3 (*p* < 0.001). All results display significant correlations based on 95% confidence intervals calculated from the bootstrap procedure; for further details, including nonsignificant results, see Supplementary Materials Figure S4 [Color figure can be viewed at http://wileyonlinelibrary.com]

The first LV delineated a functional network connected to right amygdala (AC: *r* = 0.57, 95% CI [0.44, 0.77]; AI: *r* = 0.47, 95% CI [0.25, 0.78]; HC: *r* = 0.64, 95% CI [0.55, 0.83]; HI: *r* = 0.48, 95% CI [0.29, 0.71]), whose activity positively correlated with FA in the ILF during congruent anger (*r* = 0.44, 95% CI [0.23, 0.65]) and with RTs during incongruent anger (*r* = 0.32, 95% CI [0.07, 0.61]), and negatively correlated with RTs during conditions involving the happy emotion videos (both congruent (*r* = −0.29, 95% CI [−0.60, −0.12] and incongruent (*r* = −0.32, 95% CI [−0.61, −0.12])). This finding suggests that the *faster* the recognition of *happiness* is, the stronger the activity in this network, and that functional connectivity in this network is associated with greater FA in the ILF during *anger congruent* with cue (see Figure 2). This network involved left inferior frontal gyrus, right medial and superior frontal gyri, left superior temporal gyrus, left thalamus, right amygdala extending into the hippocampus, right posterior cingulate cortex, and left lingual gyrus (see Table 1).

**Table 1:** Functional connections with right amygdala

			MNI coordinates				
Brain region	Hem	BA	*x*	*y*	*z*	Voxels	BSR
*LV1*							
Inferior frontal gyrus	L	47	−38	30	−16	204	5.92
Medial frontal gyrus	R	8	12	50	30	156	4.73
Superior frontal gyrus	R	9	2	60	18	106	4.46
Amygdala extending into the hippocampus	R		22	−8	−18	3,336	6.93
Posterior cingulate cortex	R	30	18	−56	12	160	5.33
Parahippocampus	R	19	22	−50	−4	107	5.04
Thalamus	L		−28	−22	8	247	6.27
Superior temporal gyrus	L	22	−52	−12	0	5,991	8.00
Lingual gyrus	L	18	−8	−58	6	141	5.15
*LV2*							
sACC	L	32	−6	24	−14	110	5.69
Mammillary bodies extending into the hippocampus	R		10	−14	−20	124	5.08
Caudate	L		−8	8	16	136	5.02

Abbreviations: Hem = hemisphere; BA = Brodmann area; R = right; L = left; BSR = bootstrap ratio; subgenual anterior cingulate cortex (sACC); voxels = number of voxels (one voxel volume = 6 mm^3^). All reported activations are from TR 3 and 5 ≥100 voxels (600 mm^3^).

#### 4.1.2 LV2: right amygdala network, stria terminalis, and faster response to congruent anger

**Figure 3:**
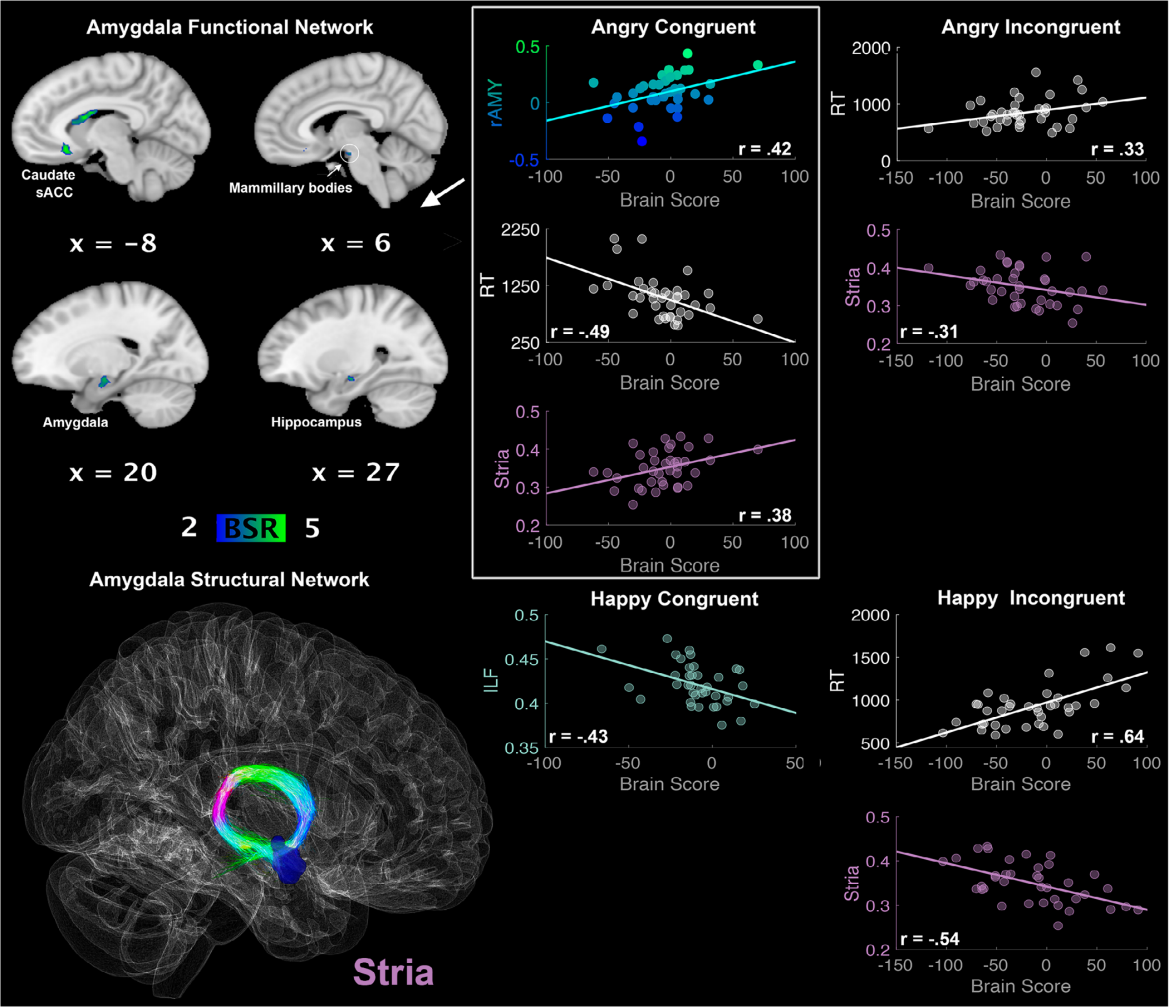
LV2: functional connections with right amygdala (rAMY) and structural/behaviour PLS results. (Top left panel) A pattern of correlated whole‐brain activity, (bottom left panel) structural network: right stria terminalis (stria). (Top right panel) During angry conditions, correlations between activity in rAMY seed and brain scores representing network activity displayed in the top left panel, with positive correlation for stria FA and negative correlation for reaction times during congruent anger conditions. Negative correlation for stria FA and positive correlation for reaction times during incongruent angry conditions. (Bottom right panel) Negative correlation for stria FA and positive correlation for reaction times during incongruent happy conditions. rAMY seed activity is displayed in percent signal change and reaction times are displayed in milliseconds. BSR threshold is set at 2 (*p* < 0.05), for visualisation purposes; however, reported whole‐brain activity is set at BSR 3 (*p* < 0.001). All results display significant correlations based on 95% confidence intervals calculated from the bootstrap procedure; for further details, including nonsignificant results, see Supplementary Materials Figure S5 [Color figure can be viewed at http://wileyonlinelibrary.com]

Critically, the second LV delineated a functional network connected to right amygdala (*r* = 0.42, 95% CI [0.21, 0.81]) whose activity negatively correlated with RTs (*r* = −0.49, 95% CI [−0.75, −0.42]) during congruent angry conditions and positively correlated with FA in the stria terminalis (*r* = 0.38, 95% CI [0.34, 0.62]). In other words, the *faster* the recognition of *anger congruent* with cue is, the stronger the activation and microstructure of this functional and structural network, respectively (see Figure 3). This network involved right amygdala and hippocampus extending into the mammillary bodies, caudate, and subgenual anterior cingulate cortex (see Table 1). People who recruited this network of regions also had reduced FA in the stria terminalis (AI: *r* = −0.31, 95% CI [−0.55, −0.12]; HI: *r* = −0.54, 95% CI [−0.70, −0.46]) and slower RTs (AI: *r* = 0.33, 95% CI [0.21, 0.67]; HI: *r* = 0.64, 95% CI [0.56, 0.82]) during the incongruent conditions (both anger and happiness).

### 4.2 Structure–function–behaviour relation between right temporoparietal junction and IFOF

The analysis with rTPJ identified two statistically significant LVs (*p* < 0.01), accounting for 20.95% and 13.25% of covariance in the data.

#### 4.2.1 LV1: right TPJ network, IFOF, and slower response to congruent anger

**Figure 4:**
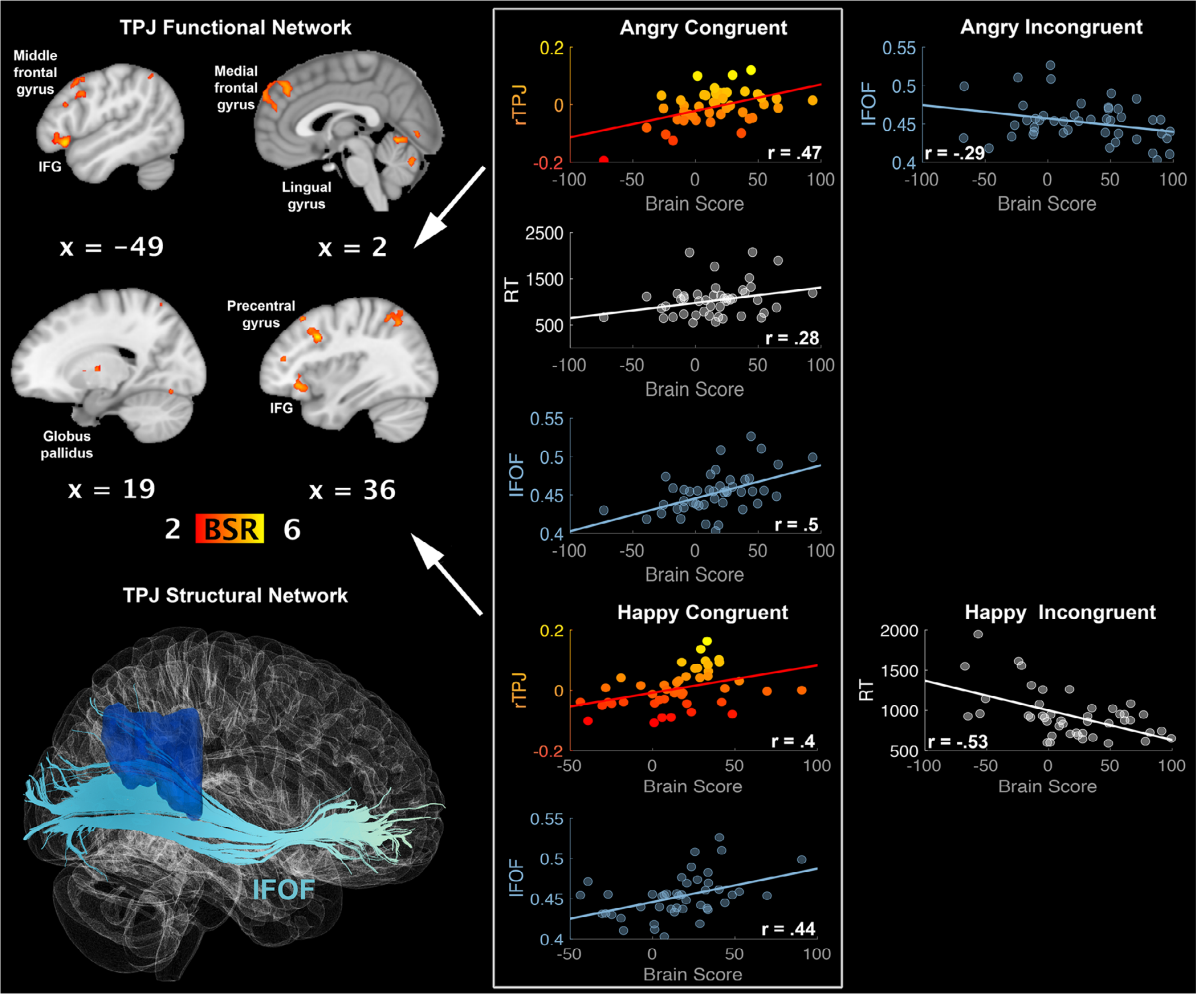
LV1: functional connections with right temporoparietal junction (rTPJ) and structural/behaviour PLS results. (Top left panel) A pattern of correlated whole‐brain activity, (bottom left panel) structural network: right inferior fronto‐occipital fasciculus (IFOF). (Top right panel) During angry congruent conditions, correlations between activity in rTPJ seed and brain scores representing network activity displayed in the top left panel, with positive correlation for FA in the IFOF, and positive correlation for reaction times. (Bottom right panel) During happy congruent conditions, correlations between activity in rTPJ seed and brain scores representing network activity displayed in the top left panel, with positive correlation for FA in the IFOF. rTPJ seed activity is displayed in percent signal change and reaction times are displayed in milliseconds. BSR threshold is set at 2 (*p* < 0.05), for visualisation purposes; however, reported whole‐brain activity is set at BSR 3 (*p* < 0.001). All results display significant correlations based on 95% confidence intervals calculated from the bootstrap procedure; for further details, including nonsignificant results, see Supplementary Materials Figure S6 [Color figure can be viewed at http://wileyonlinelibrary.com]

The first LV demonstrated a pattern of rTPJ functional connectivity (AC: *r* = 0.47, 95% CI [0.26, 0.74]; HC: *r* = 0.40, 95% CI [0.30, 0.64]), which also correlated positively with FA in the IFOF during both angry (*r* = 0.50, 95% CI [0.34, 0.77]) and happy (*r* = 0.44, 95% CI [0.32, 0.71]) congruent conditions. The rTPJ functional network, during congruent angry conditions, also correlated positively with RTs (*r* = 0.28, 95% CI [0.17, 0.49]); meaning that the *slower* the recognition of *anger congruent* with cue is, the stronger the activation of this network (see Figure 4). This network included the left middle and medial frontal gyrus, bilateral inferior frontal gyri, left superior parietal lobule, right globus pallidus, right middle temporal gyrus, and left lingual gyrus (see Table 2).

**Table 2:** Functional connections with right temporoparietal junction

			MNI coordinates				
Brain region	Hem	BA	*x*	*y*	*z*	Voxels	BSR
*LV1*							
Precentral gyrus	R	9	38	14	36	807	6.82
Middle frontal gyrus	L	6, 46	−34	20	44	366	5.80
Inferior frontal gyrus	B	47, 46	−54	32	−10	190	5.68
			34	30	−8	101	5.28
Medial frontal gyrus	L	8	−6	36	38	646	5.29
Globus pallidus	R		16	0	4	118	4.85
Middle temporal gyrus	R	21	62	−48	−2	324	6.13
Supramarginal gyrus	R	40	58	−52	34	659	5.74
Superior parietal lobule	L	7	−30	−62	56	450	5.40
Lingual gyrus	L	18	−10	−94	−10	381	4.81
*LV2 network 1*							
Inferior frontal gyrus	R	10	44	52	−14	119	5.40
Supramarginal gyrus	R	40	60	−48	34	164	10.76
Superior parietal lobule	L	7	−34	−64	58	161	4.68
Precuneus	R	7	4	−48	56	250	6.23
Paracentral lobule	L	5	−20	−34	48	138	4.33
Cuneus	R	19	6	−78	42	115	3.97
*LV2 network 2*							
Inferior frontal gyrus	L	47, 9	−40	26	−4	380	6.38
Precentral	L	9	−40	18	34	124	6.04
Anterior cingulate cortex	B	32, 24	−4	42	2	114	4.95
			10	34	−2	283	4.30
Middle frontal gyrus	R	46	54	26	20	122	4.77
Amygdala	R		30	−12	−14	102	5.57
Claustrum	L		−34	8	−2	396	6.20
Caudate	L		−8	20	14	103	5.78
Red nucleus	L		−4	−24	−18	111	5.24
Thalamus	B		14	−24	12	254	5.09
			−20	−12	18	121	4.36
Substania Nigra	L		−12	−20	−16	202	4.84
Globus pallidus	R		24	−10	−4	270	4.67
Middle temporal gyrus	L	20	−56	−40	−8	107	5.56

Abbreviations: Hem = hemisphere; BA = Brodmann area; R = right; L = left; B = bilateral; TPJ = temporoparietal junction; BSR = bootstrap ratio; voxels = number of voxels (one voxel volume = 6 mm^3^). All reported activations are from TR 1–5 ≥100 voxels (600 mm^3^).

#### 4.2.2 LV2: two right TPJ networks differentially associated with (1) faster response to incongruent anger and slower response to congruent anger versus (2) faster response to congruent happiness

**Figure 5:**
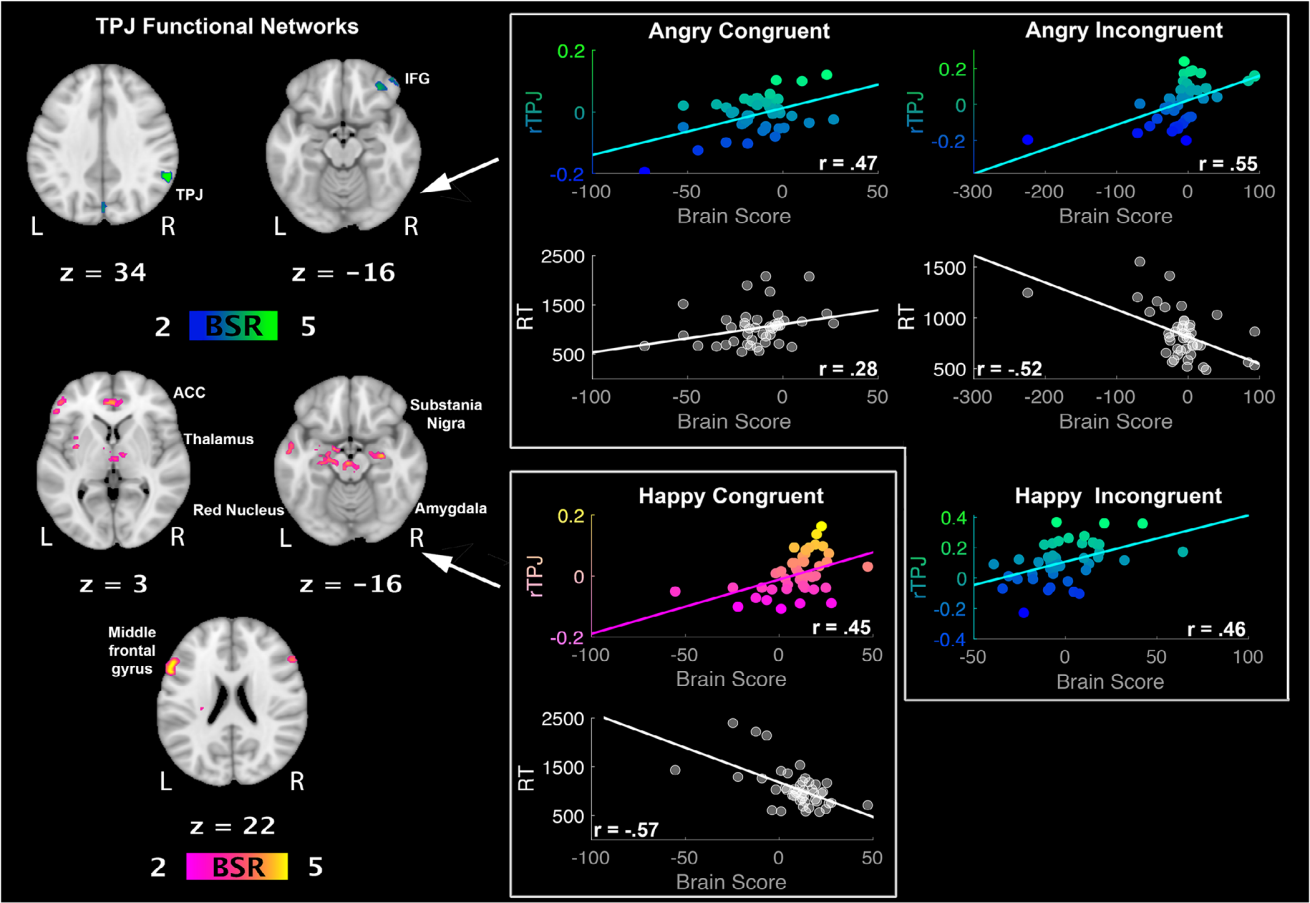
LV2: functional connections with right temporoparietal junction (rTPJ) and structural/behaviour PLS results. (Left panel) Two alternative patterns of correlated whole‐brain activity. (Top right panel) During angry conditions, correlations between activity in rTPJ seed and brain scores representing network activity displayed in the top left panel, with positive correlation for reaction times during congruent conditions, and negative correlation for reaction times during incongruent conditions. (Bottom right panel) Correlations between activity in rTPJ seed and brain scores representing network activity displayed in the bottom left panel, with negative correlation for reaction times during congruent happy conditions. rTPJ seed activity is displayed in percent signal change and reaction times are displayed in milliseconds. BSR threshold is set at 2 (*p* < 0.05), for visualisation purposes; however, reported whole‐brain activity is set at BSR 3 (*p* < 0.001). All results display significant correlations based on 95% confidence intervals calculated from the bootstrap procedure; for further details, including nonsignificant results, see Supplementary Materials Figure S7 [Color figure can be viewed at http://wileyonlinelibrary.com]

Importantly, the second LV delineated a functional network connected to rTPJ (AC: *r* = 0.47, 95% CI [0.19, 0.76]; AI: *r* = 0.55, 95% CI [0.47, 0.72]; HI: *r* = 0.46, 95% CI [0.31, 0.68]), whose activity negatively correlated with RTs during incongruent angry conditions (*r* = −0.52, 95% CI [−0.73, −0.45]); meaning that the *faster* the recognition of *anger incongruent* with cue is, the stronger the activation of this network. This same network was positively correlated with RTs during congruent anger (*r* = 0.28, 95% CI [0.07, 0.55]), meaning that this network is involved in *slower* recognition of *anger congruent* with cue (see Figure 5). This network involved right inferior frontal gyrus and supramarginal gyrus, left superior parietal lobule, and right cuneus (see Table 2). Interestingly, an alternative rTPJ network was engaged during happy congruent conditions (*r* = 0.45, 95% CI [0.35, 0.71]) and negatively correlated with RTs (*r* = −0.57, 95% CI [−0.84, −0.47]). This network involved left inferior frontal gyrus, right middle frontal gyrus, bilateral anterior cingulate cortex, left middle temporal gyrus, bilateral thalamus, right amygdala, left caudate, red nucleus, substania nigra, and right globus pallidus (see Table 2 and Figure 5).

## 5 DISCUSSION

The current study identified functional and structural networks that facilitate speed of emotion processing, in situations either congruent or incongruent with prior expectations. We identified a role for the stria terminalis, a white‐matter pathway connecting the extended amygdala network, in emotion processing. Specifically, we found that greater FA in the right stria terminalis was associated with faster response to emotion during actual presentation of, or after cueing, anger. Greater FA in the stria terminalis pathway was also associated with stronger connectivity in a rAMY‐limbic functional network, and individuals with greater recruitment of this functional network responded faster to *anger congruent* with cue. In contrast, faster response to *anger incongruent* with cue was associated with a ventral frontoparietal network, which was functionally connected to the rTPJ. Furthermore, we found that a widespread network, involving frontotemporal and subcortical regions, which was functionally connected to both the rAMY and rTPJ, was associated with faster response times for happy videos. In sum, we provide evidence that the extended amygdala circuitry facilitates speed of response in threatening situations and, for the first time, highlight a key role for the stria terminalis in the efficient recognition of emotion under threat. Furthermore, we advance on current knowledge by demonstrating differential influence of the rAMY‐limbic and rTPJ‐ventral attention network on fast processing of anger, depending on if anger is congruent or incongruent with prior expectations, respectively.

We found that both functional and structural connectivity within the extended amygdala network facilitate speed of emotion processing in threatening conditions. The extended amygdala network includes the amygdala and the BNST. Evidence suggests that these two regions have distinct roles in threat perception, specifically phasic versus sustained response (Alvarez, Chen, Bodurka, Kaplan, & Grillon, 2011; Herrmann et al., 2016; Naaz, Knight, & Depue, 2019). The most recent paper by Naaz et al. (2019) found that the amygdala is involved in phasic responses to *explicit* (cued) threat and the BNST involved in sustained responses to *ambiguous* threat. In line with the results from Naaz et al. (2019), we found that recognition of *explicitly cued* anger was faster in those participants who had stronger functional connectivity with the right amygdala. The stria terminalis links these two regions within the extended amygdala network, connecting the amygdala to the BNST. It is not surprising then that our findings show that individuals who have greater FA in the stria terminalis recognised anger faster when it was *both* congruent and incongruent with the cue. No prior study has investigated the stria terminalis and its association with human social behaviour; however, previous studies have linked the stria terminalis with traits that impair threat processing. For example, FA in the stria terminalis has been found to be related with high levels of anxiety sensitivity in patients with panic disorder (Kim, Kim, Choi, & Lee, 2017); similarly, variability in the microstructure of the stria terminalis was found to vary with post‐traumatic stress severity (Harnett, Ference, Knight, & Knight, 2018). FA in the stria terminalis was also found to differ across levels of consciousness in patients with disorders of consciousness (Wu et al., 2018). The current study provides novel evidence that the stria terminalis is related to speed of emotion processing in threatening or potentially threatening social situations, which may explain the association with anxiety‐related disorders.

We found a strong association between the stria terminalis and the functional rAMY‐limbic network, demonstrating that the increased microstructure and, subsequently, the ability for regions within the network to communicate are crucial for faster processing in threatening situations. To date, the role of the stria terminalis in human cognition has been difficult to study due to the difficulty in tracing the tract. The use of conservative deterministic tractography and manual tracing of tracts, and the advancement of diffusion imaging (e.g., using HARDI acquisition protocol) have given us the possibility to trace the stria terminalis with high confidence. The stria terminalis tract connects the AMY with the caudate, thalamus, hippocampus, and septal and hypothalamic nuclei (Avery et al., 2014; Kwon, Byun, Ahn, Son, & Jang, 2011). In the current study, we found high concordance between regions connected by the stria terminalis and the regions within the rAMY‐limbic functional network, which included: caudate, hippocampus, mammillary bodies, and subgenual anterior cingulate cortex (sACC). Theories pertaining to the role of the rAMY state that it is involved in allocating processing resources to prioritise cues relevant in a given situation (Pessoa & Adolphs, 2010) and, as such, would facilitate rapid detection of threat, relevant for survival (Bishop, 2008; Marstaller et al., 2016b; Vuilleumier & Pourtois, 2007). Previous studies have found that the sACC and caudate are associated with fast responses to threatening versus neutral faces, with evidence for involvement in prioritising attention towards threatening faces (Doty et al., 2014). Connectivity between the hippocampus and amygdala has been strongly associated with contextual fear conditioning, in which participants are exposed to a particular context paired with a threatening stimulus, such as a shock, resulting in an automatic fear response during that specific context (Alvarez, Biggs, Chen, Pine, & Grillon, 2008; Marstaller, Burianová, & Reutens, 2016a). Mammillary bodies, thought to be part of the extended hippocampal system, rapidly relay hippocampal inputs to the thalamus (Vann, 2010). The interplay between the rAMY and the extended hippocampal system may produce contextual fear conditioning, in which there is a focus on threatening stimuli. In summary, our findings align with the idea that memory‐encoded fear conditioning and prioritisation of attention to threatening cues underlie fast responding to anger that is congruent with prior expectations.

We found that greater engagement of an rTPJ functional network was associated with faster recognition of anger incongruent with cue. This network comprised of right inferior frontal gyrus, right precuneus, right cuneus, and left superior parietal lobule connecting to the rTPJ, part of the ventral attention network (Corbetta, Kincade, Ollinger, McAvoy, & Shulman, 2000; Corbetta & Shulman, 2002; Kincade, Abrams, Astafiev, Shulman, & Corbetta, 2005). This ventral attention network specialises in bottom‐up processing, interrupting top‐down attention and shifting focus to unexpected, but relevant stimuli—such as unexpected threat (Corbetta et al., 2008). Moreover, we found that individuals who engaged this rTPJ ventral attention network more had slower recognition of anger congruent with cue, which may point to inefficient inhibition of the reorienting network during congruent trials (DiQuattro & Geng, 2011). Individuals who recruited the rTPJ ventral and dorsal attention networks together during viewing of congruent emotions had increased white matter microstructure in the IFOF, suggesting that the IFOF may connect both ventral and dorsal networks. Contrary to our hypothesis, we did not find evidence that individuals who engaged the rTPJ ventral attention network more, *and* had faster responses to anger incongruent with cue, had increased FA in the IFOF. However, we found that the rTPJ structural network is important for the perception of emotions congruent with cue. Future studies should examine other structural networks of the rTPJ, such as the superior longitudinal fasciculus III (Hattori et al., 2017), to elucidate the white matter pathways involved in fast re‐orienting to unexpected, but relevant, stimuli.

One limitation in the present study is that despite the high resolution of our DWI there is a chance that fibres external to the stria terminalis have been incorrectly classified. Previous studies have traced the stria terminalis using similar voxel resolutions to ours, with a single‐shell sequence, using DTI acquisition (Kamali et al., 2015; Kwon et al., 2011; Mori & Aggarwal, 2014). However, HARDI fibre tracking (used in this study) has been found to perform significantly better than DTI at following white matter tracts through regions of crossing fibres, avoiding erroneous tracing, and enabling better discrimination of more complex white matter pathways, such as stria terminalis (Berman, Lanza, Blaskey, Edgar, & Roberts, 2013). Second, we distinguished the stria terminalis from the fornix by use of a right amygdala seeding ROI. The stria terminalis reaches the amygdala, whereas the fornix terminates in the hippocampus (Kamali et al., 2015; Kwon et al., 2011). As such, we only included tracts that reach the amygdala in our analyses. A further limitation is that the results pertain only to a male population. The male sample was recruited in order to reduce heterogeneity, as males and females may differ in their emotion perception (Lambrecht et al., 2014; Stevens and Hamann, 2012). The generalizability of our findings to females should be the focus of follow‐up studies.

The results from the current study provide important insights into our neuroscientific understanding of emotion processing, which can elucidate the pathogenesis of psychiatric disorders in which impaired emotion processing is a key feature. In psychosis, for example, there is a deficit in recognising threatening emotions (Behere, 2015; Mandal, Pandey, & Prasad, 1998; van't Wout et al., 2007) and also misattribution of threat (Premkumar et al., 2008). In contrast, in anxiety and post‐traumatic stress disorder, there is an abnormal anticipation of threat (Gross & Hen, 2004). These abnormalities in threat processing have strong links to neurobiological factors; specifically, abnormal activation and connectivity in similar brain regions as those identified in the current study (i.e., amygdala, temporoparietal junction, BNST, ACC, and hippocampus) (Bitsch, Berger, Nagels, Falkenberg, & Straube, 2018; Bryant et al., 2008; Buff et al., 2017; Felmingham et al., 2008; Rabellino et al., 2015; Rabellino et al., 2018; Underwood, Kumari, & Peters, 2016; Underwood, Peters, & Kumari, 2015). In contrast to disorders of threat processing, depression has been consistently linked with impaired perception of happiness (Bourke, Douglas, & Porter, 2010) and reduced activity in the limbic network (Lawrence et al., 2004), with antidepressant treatment associated with increased amygdala activation to happy faces (Norbury et al., 2009). In our study, we found that individuals who recruited a widespread frontotemporal‐subcortical network had faster response recognition for happy stimuli. Specifically, when this network is functionally connected to the rAMY, this is related to faster recognition of happy emotions congruent or incongruent with cue, and when this network is functionally connected to the rTPJ, this is related to faster recognition of happy emotions congruent with cue. A multimodal, network level approach, such as the one used in the current study, provides greater insight into the neural architecture of healthy emotion perception, with implications for understanding a range of clinical pathologies characterised by impaired emotion processing. The findings from the current study, in combination with future research into impaired emotion processing networks, provide impetus for new neuromodulation therapies, which rely on isolating abnormal neuroanatomic networks (Tye, Frye, & Lee, 2009).

## 6 CONCLUSIONS

The results of the present study confirmed that faster detection of anger *congruent* with prior expectations is associated with greater connections within the rAMY limbic network. In threatening environments, attention is rapidly directed to cues that signal aggression, inducing connections between limbic structures and rAMY. The present study provides novel evidence as to the role of stria terminalis in emotion processing. We find that increased white matter microstructure in this tract is important for faster processing of both congruent and incongruent anger, as well as faster detection of happiness in situations where anger is cued. In terms of speed of detecting anger *incongruent* with prior expectations, we found involvement of the ventral attention network with a primary node of the rTPJ, which is known to interrupt top‐down attention and shift focus to unexpected, behaviourally relevant stimuli. Our results have implications for understanding the neurobiological underpinnings of psychiatric disorders where there is an impaired processing of emotion and may inform the application of neuromodulation therapies for these psychiatric disorders.

## CONFLICT OF INTERESTS

The authors declare no competing financial interests.

## Supporting information


**Fig. S1 The Stria Terminalis.** Left panel: individual stria termialis tracts from 3 randomly selected participants displayed on their T1s. Top right panel: inclusion and exclusion ROIs for the stria terminalis. Bottom right panel: the stria terminalis tract compared to the fornix (from the FSL atlas [Brown, et al., 2017]), highlighting that the fornix does not reach the amygdala but the stria terminalis does reach the amygdala.
**Figure S2. The Inferior Longditudinal Fasciculus (ILF).** Left panel: individual ILF tracts from 3 randomly selected participants displayed on their T1s. Top right panel: inclusion ROIs for the ILF. Bottom right panel: the ILF tract.
**Figure S3. The inferior fronto‐occipital fasciculus (IFOF).** Left panel: individual IFOF tracts from 3 randomly selected participants displayed on their T1s. Top right panel: inclusion ROIs for the IFOF. Bottom right panel: the IFOF tract.
**Figure S4. LV1: Functional connections with rAMY, and structural/behaviour PLS results.** (A) Correlations between activity in right amygdala (rAMY), reaction times (RTs), right stria terminalis FA (Stria), and right inferior longditudinal fasciculus FA (ILF) and activity in widespread frontotemporal limbic network. Error bars denote 95% confidence intervals for the correlations calculated from the bootstrap procedure. The significant correlations are coloured and the non‐significant correlations are grey. (B) The correlations from ‘A’ displayed in individual scatterplots.
**Figure S5. LV2: Functional connections with rAMY, and structural/behaviour PLS results.** (A) Correlations between activity in right amygdala (rAMY), reaction times (RTs), right stria terminalis FA (Stria), and right inferior longditudinal fasciculus FA (ILF) and activity in a limbic network. Error bars denote 95% confidence intervals for the correlations calculated from the bootstrap procedure. The significant correlations are coloured and the non‐significant correlations are grey. (B) The correlations from ‘A’ displayed in individual scatterplots.
**Figure S5. LV1: Functional connections with rTPJ, and structural/behaviour PLS results.** (A) Correlations between activity in right temporoparietal junction (rTPJ), reaction times (RTs), and right inferior fronto‐occipital fasciculus FA (IFOF) and activity in a dorsal attention network. Error bars denote 95% confidence intervals for the correlations calculated from the bootstrap procedure. The significant correlations are coloured and the non‐significant correlations are grey. (B) The correlations from ‘A’ displayed in individual scatterplots.
**Figure S5. LV2: Functional connections with rTPJ, and structural/behaviour PLS results.** (A) Correlations between activity in right temporoparietal junction (rTPJ), reaction times (RTs), and right inferior fronto‐occipital fasciculus FA (IFOF) and activity in a ventral attention network (blue to green) vs. limbic network (pink to yellow). Error bars denote 95% confidence intervals for the correlations calculated from the bootstrap procedure. The significant correlations are coloured and the non‐significant correlations are grey. (B) The correlations from ‘A’ displayed in individual scatterplots.

## Data Availability

The article's supporting data and materials have been made available; please find example video stimuli here: doi:10.14264/uql.2017.120.
