# Supplementary material for: Stria terminalis, amygdala, and temporoparietal junction networks facilitate efficient emotion processing under expectations
Source: Hum Brain Mapp. 2019 Aug 28;40(18):5382–96. doi: 10.1002/hbm.24779 (PMC6864902; doi:10.1002/hbm.24779)
Supplement: Supplementary file 1 — Fig. S1 The Stria Terminalis. Left panel: individual stria termialis tracts from 3 randomly selected participants displayed on their T1s. Top right panel: inclusion and exclusion ROIs for the stria terminalis. Bottom right panel: the stria terminalis tract compared to the fornix (from the FSL atlas [Brown, et al., 2017]), highlighting that the fornix does not reach the amygdala but the stria terminalis does reach the amygdala. Figure S2. The Inferior Longditudinal Fasciculus (ILF). Left panel: individual ILF tracts from 3 randomly selected participants displayed on their T1s. Top right panel: inclusion ROIs for the ILF. Bottom right panel: the ILF tract. Figure S3. The inferior fronto‐occipital fasciculus (IFOF). Left panel: individual IFOF tracts from 3 randomly selected participants displayed on their T1s. Top right panel: inclusion ROIs for the IFOF. Bottom right panel: the IFOF tract. Figure S4. LV1: Functional connections with rAMY, and structural/behaviour PLS results. (A) Correlations between activity in right amygdala (rAMY), reaction times (RTs), right stria terminalis FA (Stria), and right inferior longditudinal fasciculus FA (ILF) and activity in widespread frontotemporal limbic network. Error bars denote 95% confidence intervals for the correlations calculated from the bootstrap procedure. The significant correlations are coloured and the non‐significant correlations are grey. (B) The correlations from ‘A’ displayed in individual scatterplots. Figure S5. LV2: Functional connections with rAMY, and structural/behaviour PLS results. (A) Correlations between activity in right amygdala (rAMY), reaction times (RTs), right stria terminalis FA (Stria), and right inferior longditudinal fasciculus FA (ILF) and activity in a limbic network. Error bars denote 95% confidence intervals for the correlations calculated from the bootstrap procedure. The significant correlations are coloured and the non‐significant correlations are grey. (B) The correlations from ‘A’ disp [file HBM-40-5382-s001.docx]

Supplementary Information


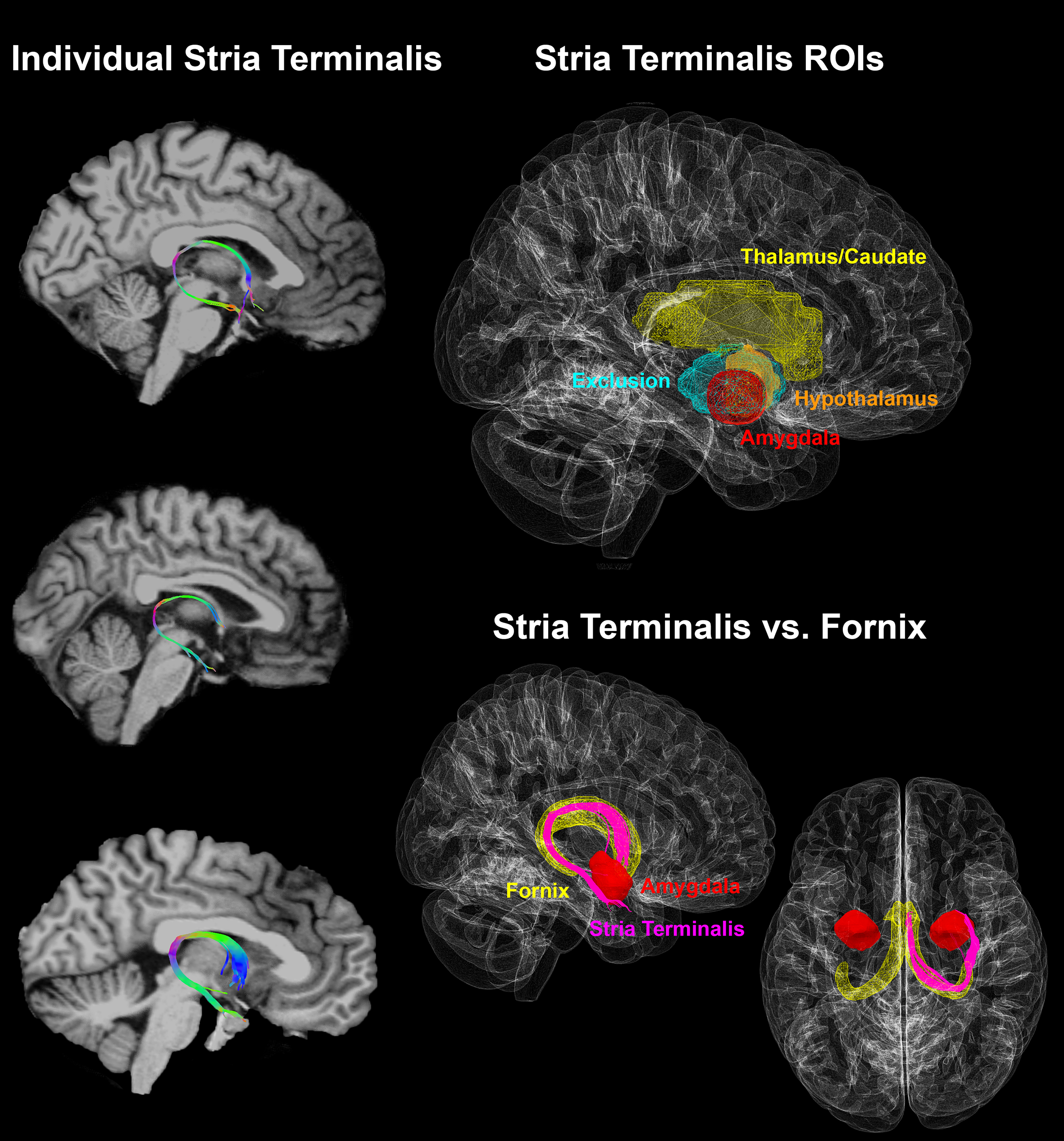


**Fig. S1. The Stria Terminalis.** Left panel: individual stria termialis tracts from 3 randomly selected participants displayed on their T1s. Top right panel: inclusion and exclusion ROIs for the stria terminalis. Bottom right panel: the stria terminalis tract compared to the fornix (from the FSL atlas (Brown, et al., 2017)), highlighting that the fornix does not reach the amygdala but the stria terminalis does reach the amygdala.


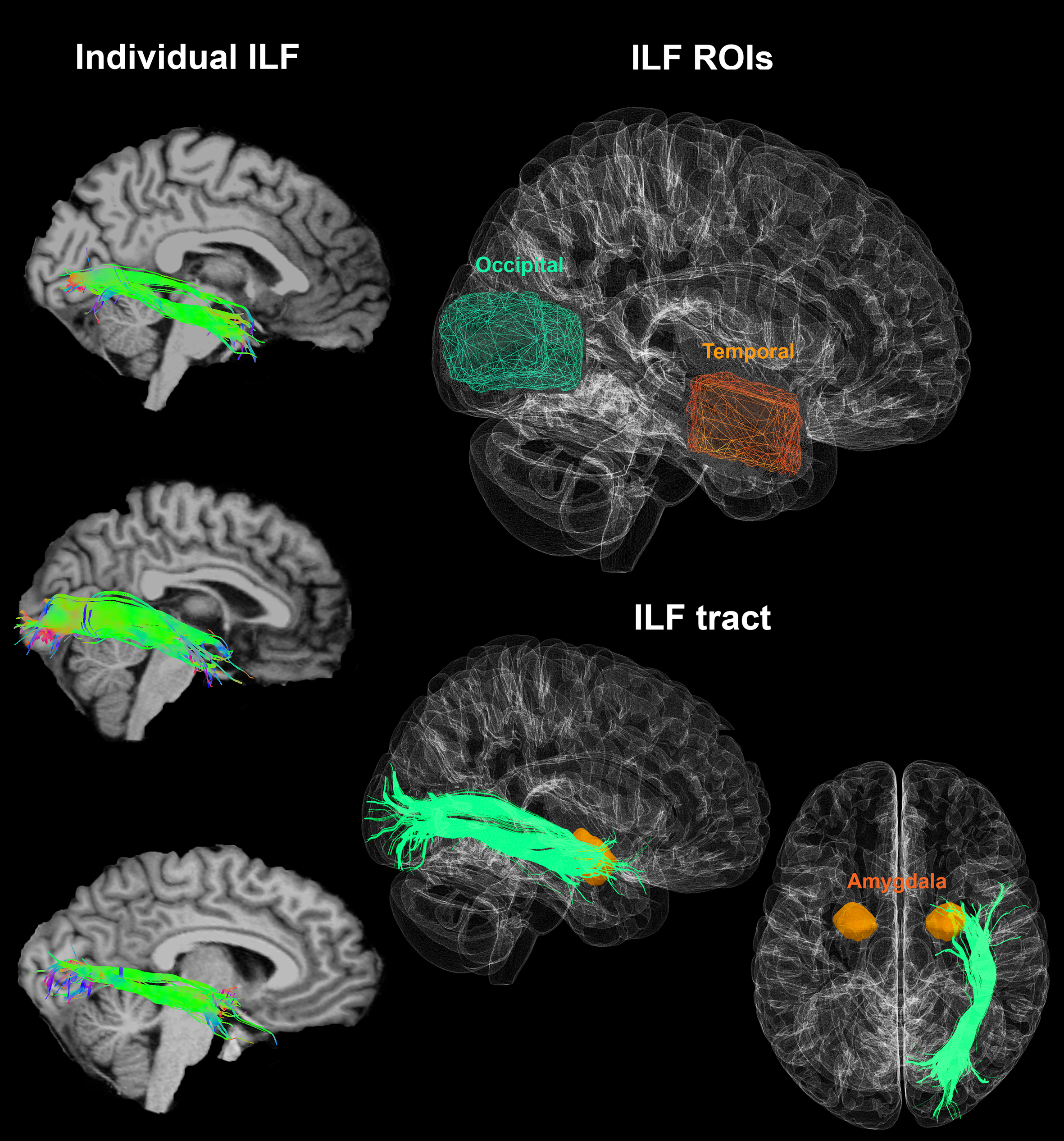


**Fig. S2. The Inferior Longditudinal Fasciculus (ILF).** Left panel: individual ILF tracts from 3 randomly selected participants displayed on their T1s. Top right panel: inclusion ROIs for the ILF. Bottom right panel: the ILF tract.


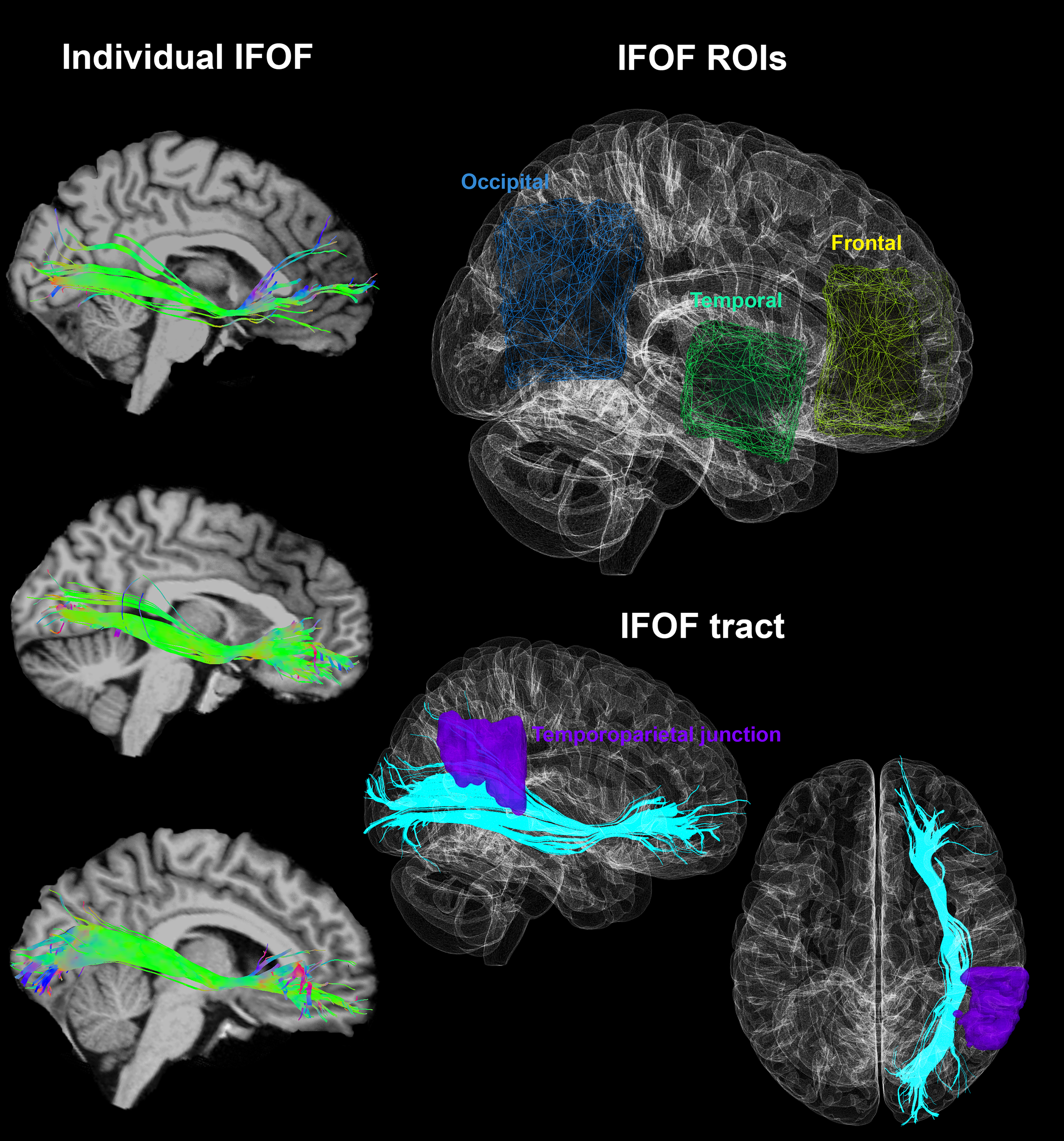


**Fig. S3. The inferior fronto-occipital fasciculus (IFOF).** Left panel: individual IFOF tracts from 3 randomly selected participants displayed on their T1s. Top right panel: inclusion ROIs for the IFOF. Bottom right panel: the IFOF tract.


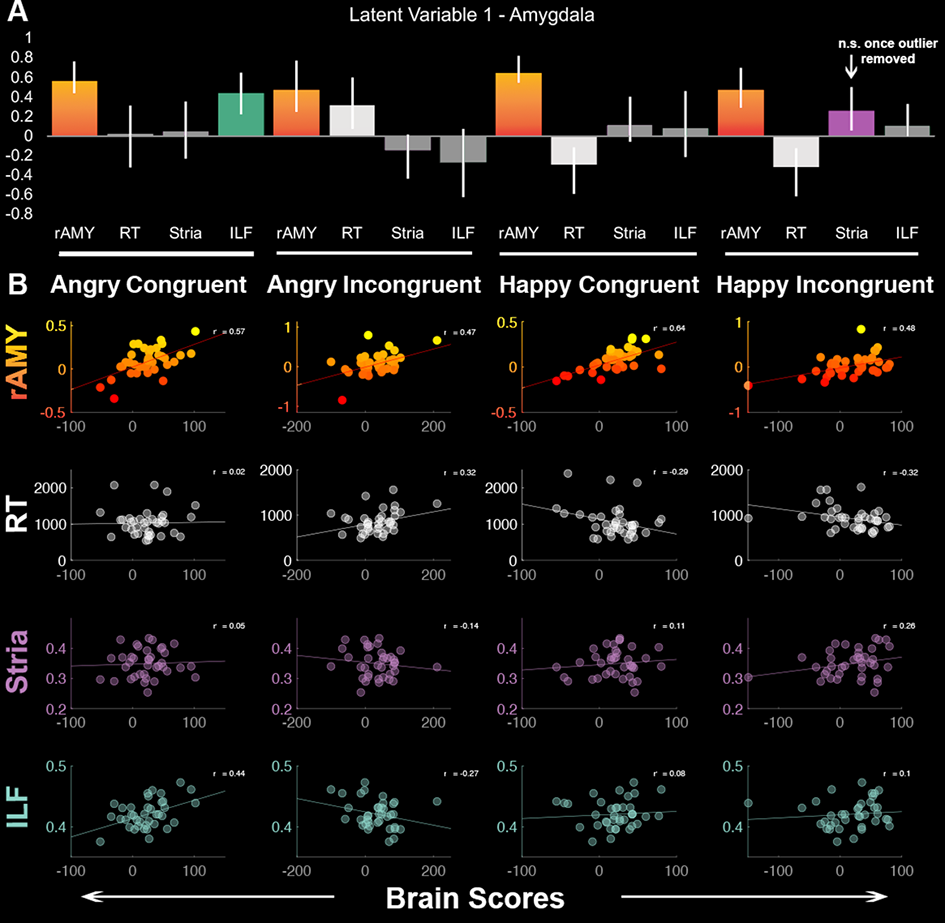


**Fig. S4. LV1: Functional connections with rAMY, and structural/behaviour PLS results.** (A) Correlations between activity in right amygdala (rAMY), reaction times (RTs), right stria terminalis FA (Stria), and right inferior longditudinal fasciculus FA (ILF) and activity in widespread frontotemporal limbic network. Error bars denote 95% confidence intervals for the correlations calculated from the bootstrap procedure. The significant correlations are coloured and the non-significant correlations are grey. (B) The correlations from ‘A’ displayed in individual scatterplots.


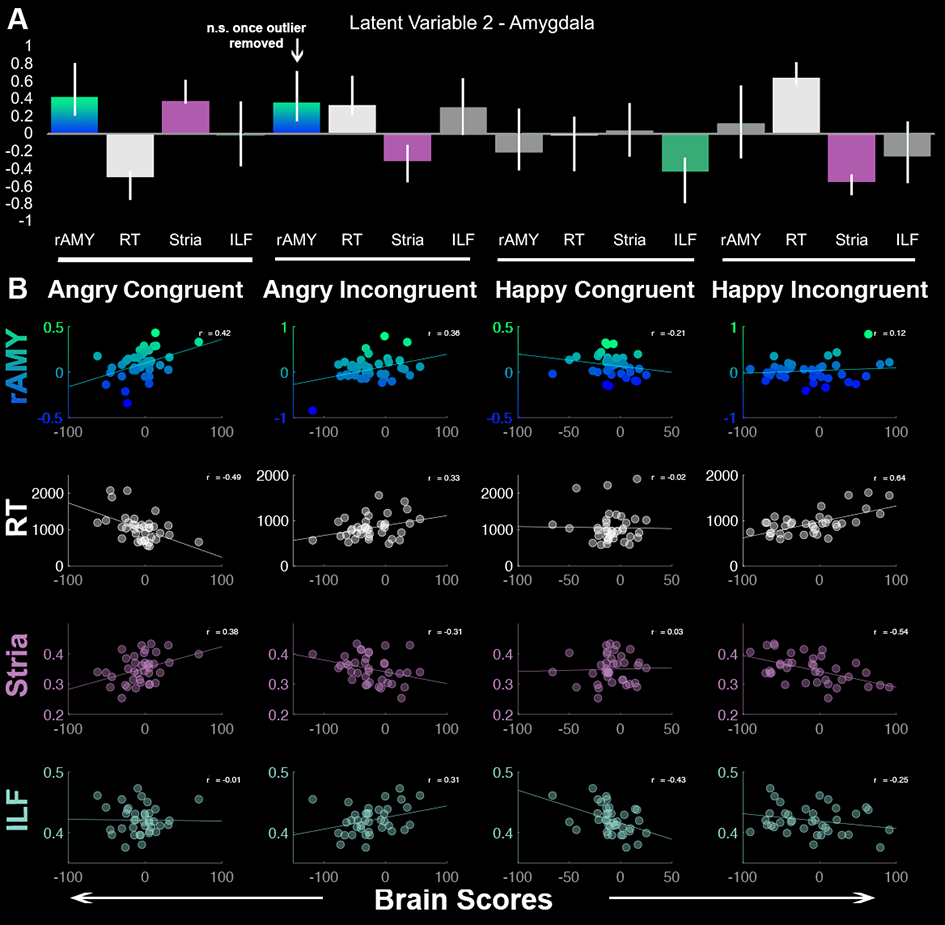


**Fig. S5. LV2: Functional connections with rAMY, and structural/behaviour PLS results.** (A) Correlations between activity in right amygdala (rAMY), reaction times (RTs), right stria terminalis FA (Stria), and right inferior longditudinal fasciculus FA (ILF) and activity in a limbic network. Error bars denote 95% confidence intervals for the correlations calculated from the bootstrap procedure. The significant correlations are coloured and the non-significant correlations are grey. (B) The correlations from ‘A’ displayed in individual scatterplots.


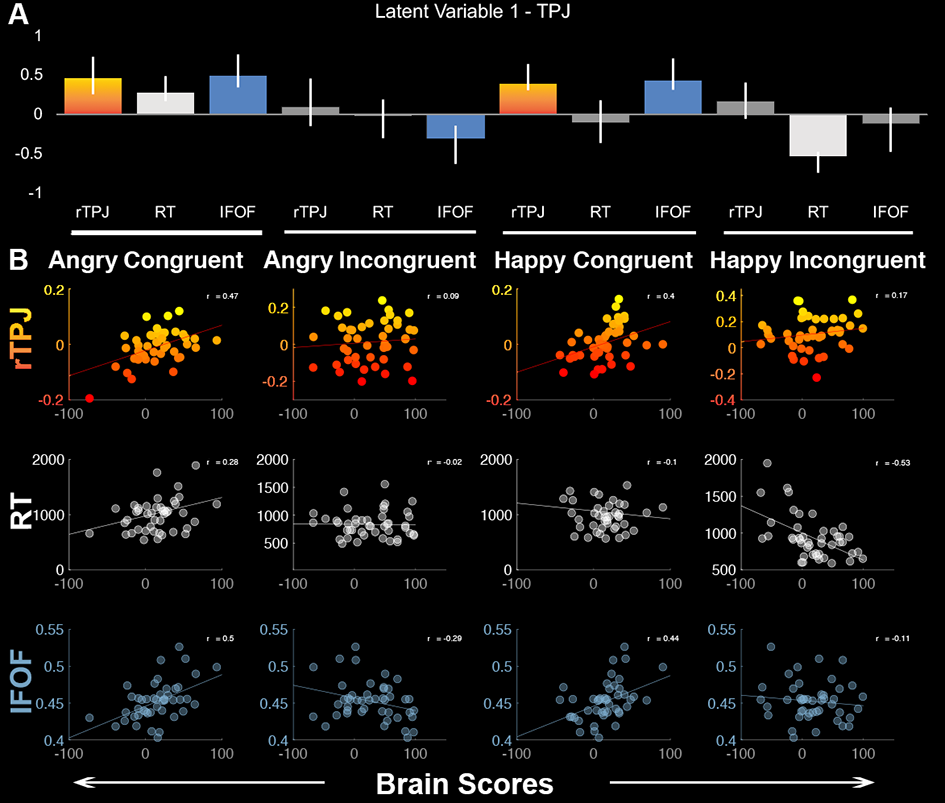


**Fig. S5. LV1: Functional connections with rTPJ, and structural/behaviour PLS results.** (A) Correlations between activity in right temporoparietal junction (rTPJ), reaction times (RTs), and right inferior fronto-occipital fasciculus FA (IFOF) and activity in a dorsal attention network. Error bars denote 95% confidence intervals for the correlations calculated from the bootstrap procedure. The significant correlations are coloured and the non-significant correlations are grey. (B) The correlations from ‘A’ displayed in individual scatterplots.


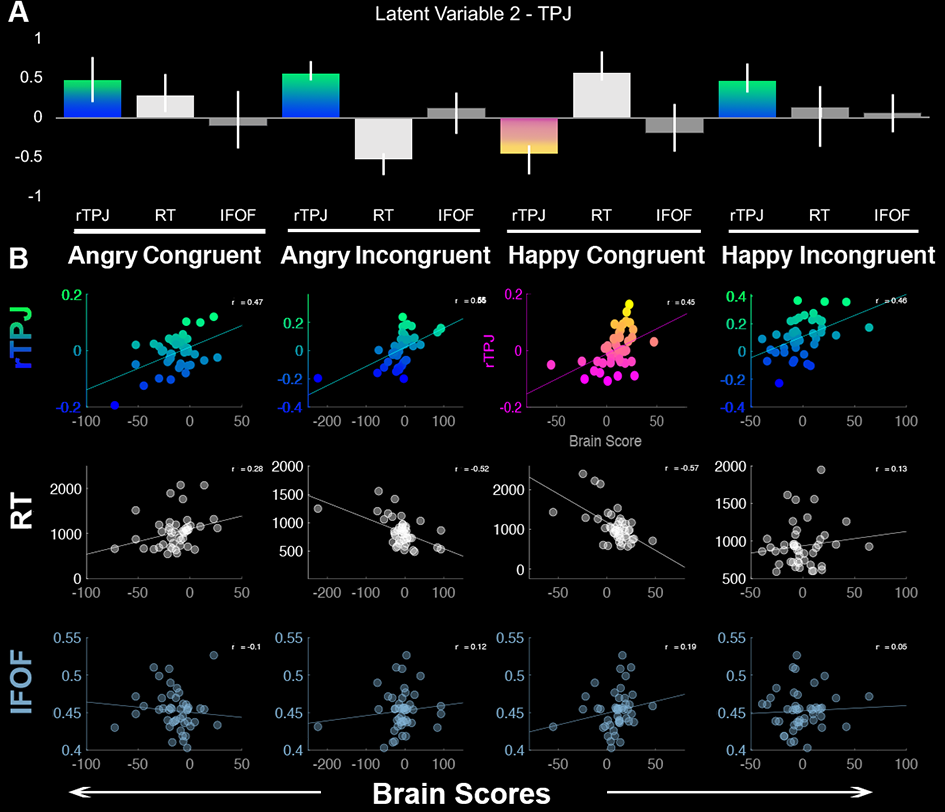


**Fig. S5. LV2: Functional connections with rTPJ, and structural/behaviour PLS results.** (A) Correlations between activity in right temporoparietal junction (rTPJ), reaction times (RTs), and right inferior fronto-occipital fasciculus FA (IFOF) and activity in a ventral attention network (blue to green) vs. limbic network (pink to yellow). Error bars denote 95% confidence intervals for the correlations calculated from the bootstrap procedure. The significant correlations are coloured and the non-significant correlations are grey. (B) The correlations from ‘A’ displayed in individual scatterplots.

**Reference**

Brown CA, Johnson NF, Anderson-Mooney AJ, Jicha GA, Shaw LM Trojanowski JQ, Van Eldik LJ, Schmitt FA, Smith CD, & Gold BT (2017). Development, validation and application of a new fornix template for studies of aging and preclinical Alzheimer’s disease. NeuroImage: Clinical, 13; 106-115.
